# Primary adenocarcinoma of the upper oesophagus

**DOI:** 10.3332/ecancer.2013.314

**Published:** 2013-05-02

**Authors:** Yash P Verma, Ashok K Chauhan, Rajiv Sen

**Affiliations:** 1 Department of Radiotherapy, PGIMS Rohtak, India; 2 Department of Pathology, PGIMS Rohtak, India

**Keywords:** oesophageal adenocarcinoma, middle oesophagus, rare, malignancy, radiotherapy

## Abstract

Upper oesophageal adenocarcinoma is a rare malignancy that occurs in the ectopic gastric mucosa, in the upper part of the oesophagus. Only 29 cases so far have been reported. We report the 30th case in a 50-year-old Asian female presenting with difficulty in swallowing.

## Introduction

Upper oesophageal malignancies (proximal to gastro-oesophageal junction) are usually of the squamous cell type. Although glandular heterotropia of the upper digestive tract is frequently observed during upper gastrointestinal endoscopy (in 0.26%–4.9% of cases), it has rare malignant potential. Adenocarcinoma of the upper oesophagus is not commonly observed and only 29 cases have been reported so far [1–8]. We are reporting on the 30th case of adenocarcinoma of the upper oesophagus in an Asian female.

## Case report

**Figure 1:**
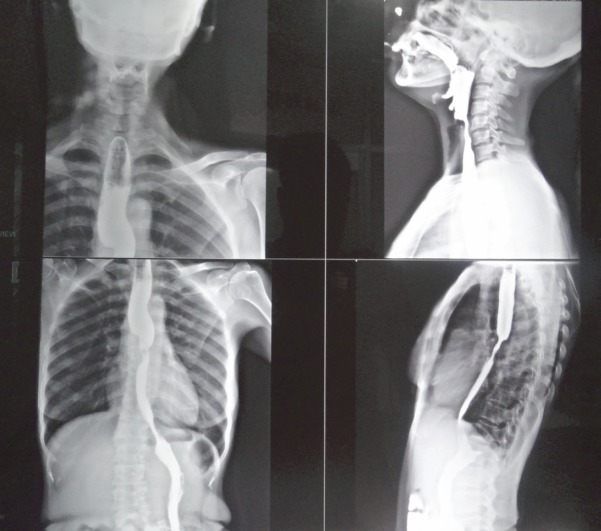
Barium swallow.

**Figure 2:**
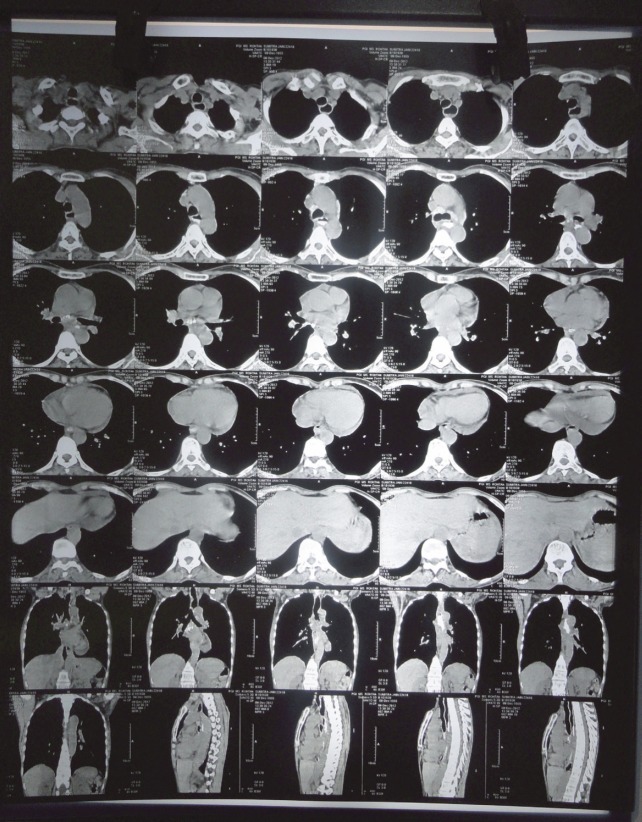
CECT Thorax.

**Figure 3:**
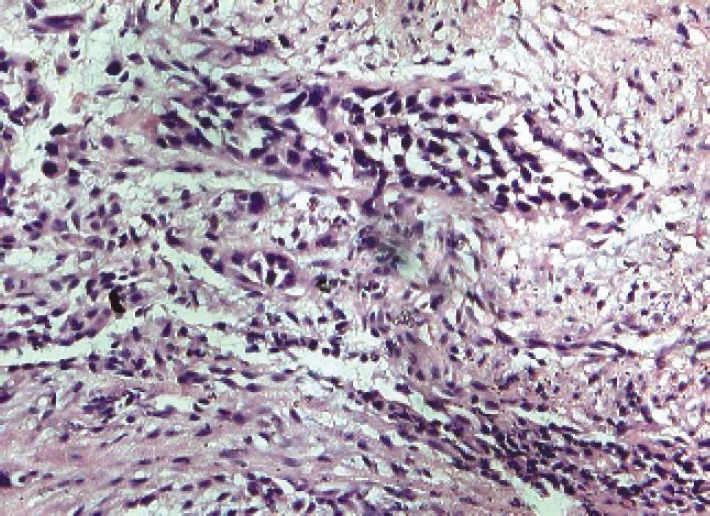
Micropictograph of biopsy specimen.

The 50-year-old patient, complained of progressive difficulty in swallowing for four months, having had only liquid intake at the time of our workup. There was no history of tuberculosis, hypertension, chronic obstructive pulmonary disease, diabetes, any other chronic ailment, or significant family history. Blood biochemistry, including liver and kidney functions, was within normal limits. Ultrasound of the patient’s abdomen was normal. A barium swallow (Figure 1) revealed a filling defect in the mid-thoracic oesophagus with shouldering effect and a hold-up of barium proximal to it. The CECT of the thorax (Figure 2) showed circumferential thickening of the oesophagus from carina to a distance of 4.5-cm. Fat plane with trachea and aorta was maintained. The gastro-oesophageal junction was not involved. The CECT of the abdomen showed normal liver, spleen, pancreas, and kidneys. No free fluid or lymphadenopathy was seen. Upper gastrointestinal endoscopy detected an ulcero-nodular, friable, obstructive growth at 22-cm from the incisor teeth; a scope negotiation below it was not possible. A biopsy (Figure 3) of growth revealed poorly differentiated adenocarcinomas. Alpha fetoprotein and serum transferrin were also within normal limits (3.1 ng/ml and 322 mg/dl). The option of surgery was given, but the patient was not willing to have any surgical intervention. In view of the moderate nutritional status and a poor oral intake, a palliative external beam radiotherapy (EBRT) was given; as 20-Gy in five fractions over five days by AP/PA fields, using telecobalt, along with supportive treatment. Oral intake and general condition improved in the following four weeks and supplementary EBRT as 12-Gy in six fractions over one week was given. Supplementary intraluminal brachytherapy was offered in view of the good response to radiotherapy, but the patient refused. At third monthly follow-up, the patient was having an adequate semisolid and occasionally solid diet. Her general condition also improved, with no additional complaints.

## Discussion

Occurrence of adenocarcinoma is rare and has been explained in the literature on the basis of ectopic gastric type mucosa in the upper part of the oesophagus. The incidence of ectopic gastric mucosa is 4.0% in males and 2.9% in females [9]. Table 1 summarizes all these cases including this case report.

The age at presentation varies from 37 to 90 years with a mean age of 63 years. Out of 30 cases, only three are female with a male to female ratio of 9:1, which is a little higher than the ratio for overall oesophageal malignancies (3.5:1) [10]. In general, radical surgery is the mainstay of treatment. Radiotherapy is a good alternative, especially when surgery is refused or not feasible. Beyond doubt, treatment modality needs to be individualized based on disease, the patient’s condition, and other parameters. The gradual increase in the number of cases of upper oesophageal adenocarcinoma warrants the standardization of treatment protocol.

## Figures and Tables

**Table 1:** Summary of cases reported to date.

Sr. no.	Year	First author	Histological type	Age (years)	Gender
1	1950	Carrie A	Adenocarcinoma	64	M
2	1969	Davis WM	Mucinous Adenocarcinoma	68	M
3	1970	Sakamoto G	Papillary adenocarcinoma	64	M
4	1974	Clemente C	Int. type adenocarcinoma	53	M
5	1985	Schmidt H	Adenocarcinoma	37	M
6	1985	Schmidt H	Adenocarcinoma	54	M
7	1986	Endo T	Adenosquamous carcinoma	73	M
8	1987	Christensen WN	PD adenocarcinoma	52	M
9	1987	Christensen WN	MD adenocarcinoma	50	M
10	1987	Danoff B	PD adenocarcinoma	43	M
11	1989	Kamiya J	MD adenocarcinoma	58	M
12	1991	Ishii K	WD adenocarcinoma	66	M
13	1993	Kubota S	Papillary adenocarcinoma	58	M
14	1994	Takagi Y	MD adenocarcinoma	85	M
15	1995	Takagi A	WD adenocarcinoma	70	M
16	1995	Sperling RM	PD adenocarcinoma	79	M
17	1996	Kammori M	WD adenocarcinoma	74	F
18	1997	Pai S	MD adenocarcinoma	60	M
19	1997	Berkelhammer C	MD adenocarcinoma	71	M
20	1997	Yamamoto S	Adenocarcinoma	90	F
21	1998	Lauwers GY	MD adenocarcinoma	57	M
22	2001	Noguchi T	WD adenocarcinoma	73	M
23	2002	Chatelaine D	PD adenocarcinoma	61	M
24	2004	Kagawa N	MD adenocarcinoma	51	M
25	2004	Abe T	WD adenocarcinoma	50	M
26	2005	Alrawi SJ	MD adenocarcinoma	60	M
27	2006	Masashi T	Adenocarcinoma	53	M
28	2010	Komori S	Adenocarcinoma	75	M
29	2011	Bard A	Adenocarcinoma	87	M
30	2012	**PRESENT CASE**	PD adenocarcinoma	50	F
